# Improving the efficacy of potato clonal micropropagation by inoculation with the rhizosphere bacteria Azospirillum baldaniorum Sp245 and Ochrobactrum cytisi IPA7.2

**DOI:** 10.18699/VJGB-22-52

**Published:** 2022-08

**Authors:** K.Yu. Kargapolova, O.V. Tkachenko, G.L. Burygin, N.V. Evseeva, A.A. Shirokov, L.Yu. Matora, S.Yu. Shchyogolev

**Affiliations:** Saratov State Vavilov Agrarian University, Saratov, Russia; Saratov State Vavilov Agrarian University, Saratov, Russia; Saratov State Vavilov Agrarian University, Saratov, Russia Institute of Biochemistry and Physiology of Plants and Microorganisms – Subdivision of the Saratov Federal Scientific Centre of the Russian Academy of Sciences, Saratov, Russia; Institute of Biochemistry and Physiology of Plants and Microorganisms – Subdivision of the Saratov Federal Scientific Centre of the Russian Academy of Sciences, Saratov, Russia; Institute of Biochemistry and Physiology of Plants and Microorganisms – Subdivision of the Saratov Federal Scientific Centre of the Russian Academy of Sciences, Saratov, Russia; Institute of Biochemistry and Physiology of Plants and Microorganisms – Subdivision of the Saratov Federal Scientific Centre of the Russian Academy of Sciences, Saratov, Russia; Institute of Biochemistry and Physiology of Plants and Microorganisms – Subdivision of the Saratov Federal Scientific Centre of the Russian Academy of Sciences, Saratov, Russia

**Keywords:** Solanum tuberosum L., Azospirillum baldaniorum Sp245, Ochrobactrum cytisi IPA7.2, plant-microbe associations, clonal micropropagation, plant growth eff icacy, adaptability, in vitro, ex vitro, Solanum tuberosum L., Azospirillum baldaniorum Sp245, Ochrobactrum cytisi IPA7.2, растительно-микробные ассоциации, клональное микроразмножение, эффективность роста растений, адаптационная способность, in vitro, ex vitro

## Abstract

Sustainable development of agriculture depends on the provision of quality seeds to the market. Inoculation with plant-growth-promoting rhizobacteria in in vitro culture can be used to improve the growth eff icacy and performance of microplants. We examined the effect of in vitro inoculation of microplants of the cultivars Nevsky and Kondor with the strains Azospirillum baldaniorum Sp245 and Ochrobactrum cytisi IPA7.2 separately and in combination. We examined the morphological variables of plant growth in in vitro culture and under ex vitro adaptation conditions; we also investigated the growth and performance of the plants in the greenhouse. The dependence of the inoculation eff icacy on potato genotype, growth stage, and inoculum composition was ascertained throughout the experiment. In vitro, A. baldaniorum Sp245 alone and in combination with O. cytisi IPA7.2 promoted the formation of roots on the microplants of both cultivars and the growth of Nevsky shoots. During plant growth ex vitro, all growth variables of the Nevsky microplants were promoted by O. cytisi IPA7.2 alone and in combination with A. baldaniorum Sp245. In both cultivars grown in the greenhouse, shoot growth was promoted in most inoculation treatments. The survival ability of the Nevsky microplants in the greenhouse increased 1.7-fold under the effect of simultaneous inoculation. Inoculation of microplants with a combination of A. baldaniorum Sp245 and O. cytisi IPA7.2 increased the number of Nevsky minitubers 1.5-fold and the number of Kondor minitubers 3.5-fold. Inoculation with the tested strains can be used to promote the growth of microplants and increase the yield of minitubers in potato seed breeding for the production of healthy planting material.

## Abstract (ru)

Устойчивое развитие сельского хозяйства зависит от обеспечения рынка качественными семенами. Инокуляция растений рост-стимулирующими ризобактериями в культуре in vitro может быть использована для повышения эффективности роста и продуктивности микрорастений при получении оздоровленного посадочного материала картофеля. Изучено влияние инокуляции in vitro штаммами Azospirillum baldaniorum Sp245 и Ochrobactrum cytisi IPA7.2 по отдельности и в консорциуме на микрорастения сортов Невский и Кондор. Оценены морфологические параметры роста растений в культуре in vitro, в условиях адаптации ex vitro, а также показатели роста и продуктивности растений в грунтовой теплице. На протяжении всего опыта была установлена зависимость эффективности бактеризации от генотипа картофеля, этапа культивирования и состава инокулята. Методом иммунофлуоресцентного анализа показано, что оба штамма бактерий успешно вступают во взаимодействие с клетками растений без антагонистического взаимного влияния. В культуре in vitro A. baldаniorum Sр245 и консорциум штаммов стимулировали образование корней на микрорастениях обоих сортов и рост побегов сорта Невский. На этапе культивирования ex vitro на все ростовые показатели микрорастений сорта Невский положительно влияла инокуляция O. cytisi IPA7.2 и консорциум штаммов. При выращивании в теплице в большинстве вариантов инокуляции стимулировался рост побегов обоих сортов. Приживаемость растений сорта Невский в теплице повысилась под действием одновременной коинокуляции в 1.7 раза. Ино- куляция микрорастений консорциумом штаммов A. baldаniorum Sр245 и O. cytisi IPA7.2 увеличивала количество мини-клубней у сорта Невский в 1.5 раза, а у сорта Кондор – в 3.5 раза. Инокуляция изученными штаммами мо- жет быть использована для стимулирования роста микрорастений и повышения урожайности мини-клубней в системе семеноводства картофеля при получении оздоровленного посадочного материала.

## Introduction

In the production of seeds of many vegetatively propagated crops, in vitro clonal micropropagation methods have been widely used (Rajasekharan, Sahijram, 2015). In the clonal micropropagation of various plant species, rhizobacteria of different taxonomic groups can be used (Orlikowska et al., 2017; Soumare et al., 2021). Among herbaceous plants, orchid (Castillo-Pérez et al., 2021), sugarcane (Oliveira et al., 2002), and some other species (Dias et al., 2009) predominate as bacterization objects. Bacterial strains capable of promoting potato microclonal growth in vitro, adaptation to ex vitro conditions, and minituber productivity have been isolated (Oswald et al., 2010). Proper selection of bacterial associates is crucial (Wang et al., 2016). Our preliminary work has shown that pure cultures of the associative rhizobacteria Azospirillum baldaniorum Sp245 and Ochrobactrum cytisi IPA7.2 promote the growth of potato microplants in vitro and ex vitro (Tkachenko et al., 2015; Burygin et al., 2019; Kargapolova et al., 2020).

Some authors have pointed out that joint inoculation of plants with two or more strains of rhizospheric plant-growthpromoting bacteria (PGPR) can be more effective than inoculation with pure cultures (Thomas et al., 2010). When using consortia of strains for inoculation, one has to see that the component cultures are compatible (Yegorenkova et al., 2016). We have previously found that for strains with different characteristics, the inoculation of microplants during growth in vitro may be important (Burygin et al., 2018).

Here we examined the efficacy of inoculation of potato (Solanum tuberosum L. cvs. Nevsky and Kondor) microplants with pure cultures of Azospirillum baldaniorum Sp245 and Ochrobactrum cytisi IPA7.2 and with their mixture. The specific aim was to use clonal micropropagation to improve the production efficacy for seeds of healthy planting material.

## Materials and methods

Growing of potato microplants in vitro. We used microplants of two middle early potato cultivars, Kondor and Nevsky. The cultivars had been obtained from the in vitro collection of the Department of Plant Breeding, Selection, and Genetics of the Faculty of Agronomy at Saratov State Vavilov Agrarian University (Saratov) and had been produced by isolation of apical meristems. The Nevsky and Kondor cultivars were used as material for study because the State Register for Selection Achievements Admitted for Usage (National List) (https:// gossortrf.ru/gosreestr/) recommends them to be grown in the Lower Volga zone. Nevsky is a domestically bred cultivar (Russian Potato Research Center, Russia), whereas Kondor is a foreign-bred one (AGRICO U.A., Netherlands).

Microcuttings with one leaf and one bud were placed in a hormone-free liquid nutrient Murashige–Skoog medium (Murashige, Skoog, 1962). Plants were grown for 30 days at a temperature of 24 °C, a humidity of 60 %, a light intensity of 60 μM/(m2 · s), and a day length of 16 h. The shoot and root morphometric variables examined were shoot length (mm), number of nodes per shoot, average root length (mm), and number of roots per shoot.

Inoculation of microcuttings. We used two rhizospheric bacterial strains – A. baldaniorum Sp245 (Baldani et al., 1983) and O. cytisi IPA7.2 (Burygin et al., 2017, 2019). Both strains were from the IBPPM RAS Collection of Rhizosphere Microorganisms (Saratov; http://collection.ibppm.ru/). Cultures were grown at 35 °C on a rotary shaker with a stirring intensity of 120 rpm until the end of the exponential growth phase (18 h) in a liquid malate medium composed as follows (g/l): Na malate, 5.0; KH2PO4, 0.4; NaCl, 0.1; MgSO4, 0.2; FeSO4·7H2O, 0.02; Na2MoO4 · 2H2O, 0.002; NH4Cl, 1.0, pH 6.8–7.0 (Döbereiner, Day, 1976). The cells were sedimented by centrifugation at 3000 g under sterile conditions and were resuspended in 0.12 M phosphate buffer (pH 7.2) containing (g/l): KH2PO4, 0.43; Na2HPO4, 1.68; NaCl, 7.2. Centrifugation was repeated twice in phosphate-buffered saline. For inoculation, 0.1 ml of cell suspension (108 cells/ ml) was added to the tubes with plants, each tube containing 10 ml of the Murashige–Skoog medium. The final cell density in the medium was 106 cells/ml.

Bacteria were inoculated separately [A. baldaniorum Sp245 on day 0 of growth (microcuttings) and O. cytisi IPA7.2 on day 15 of growth] and in combination [simultaneously on day 15 of growth or successively (A. baldaniorum Sp245 on day 0 of growth and then O. cytisi IPA7.2 on day 15 of growth)]. The control was microplants grown without bacteria.

Growing of potato microplants ex vitro. Microplants were adapted to ex vitro conditions in a phytochamber in soil-filled vessels for 20 days (temperature, 24 °C; humidity, 60 %; light intensity, 60 μM/(m2 · s); day length, 16 h). The morphometric variables analyzed were shoot length, leaf number, and leaf area.

Next, the plants were transferred to a soil-based greenhouse covered with agrotextile and were planted in a pattern of 0.4 × 0.4 m. The temperature and humidity in the greenhouse were not regulated and depended on the weather; therefore, they were stressful for the plants (the daytime temperature could rise as high as 30 °C, and humidity could drop below 60 %). The plants were watered as needed (every 3–5 days on average). Three weeks after planting and at the beginning of the budding and flowering phase, we recorded the percentage of surviving plants, the height of the plants, the numbers of shoots and leaves, and the area of the leaves. Minitubers were dug out after the vines wilted. The number and weight of minitubers per plant and the weight and diameter of each tuber were recorded

Immunofluorescence analysis. Bacteria on plant roots were identified by immunofluorescence analysis on day 30 after inoculation, by using strain-specific antibodies (Shelud’ko et al., 2010). The controls were uninoculated and inoculated roots treated with nonspecific antibodies. Nonspecific antibody sorption was blocked by 2-h incubation of root sections at room temperature in 0.05 % polyethylene glycol solution (MW 20000) in phosphate buffer. The primary antibodies were strain-specific rabbit antibodies to the LPS of A. baldaniorum Sp245 and to the LPS of O. cytisi IPA7.2 (concentration, 50 μg/ml); the secondary antibodies were tetramethylrhodamine isothiocyanate (TRITC)-labeled goat antirabbit antibodies (Abcam, USA; concentration, 1 μg/ml).

The inoculated roots of the microplants were observed with a TCS SP5 confocal microscope (Leica Microsystems, Germany) at the Simbioz Center for the Collective Use of Research Equipment in the Field of Physical-Chemical Biology and Nanobiotechnology (IBFRM RAS, Saratov).

Statistics. The experiment was repeated twice. In each experiment, three replicates of 10 plants each were used in each experimental treatment, with a total of 30 plants per treatment in each experiment. Data from all experiments were subjected to two-way analysis of variance (ANOVA) and were evaluated for a significance level p of 0.05. To test the null hypothesis, we calculated the F-test statistic (Ffact) and then determined the least significant difference (LSD0.05) between experimental treatments. Means from each experimental treatment were subjected to multiple comparison by Duncan’s test. The program package used was AGROS, a package for statistical and biometrical-genetic analysis in plant breeding and selection (version 2.09).

## Results

Effect of bacteria on growth and development of potato microclones in vitro

**Fig. 1.**
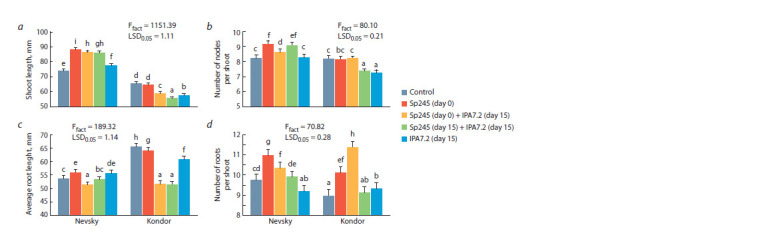
Effect of in vitro inoculation with A. baldaniorum Sp245 and O. cytisi IPA7.2 on morphological variables of potato microplants: a, shoot length; b, number of nodes per shoot; c, average root length; d, number of roots per shoot. Here and in the Figures 3–5: for all variables, a significance level p of 0.05 (n = 30) was used. Different Latin letters (a, b, c, etc.) indicate values from treatments that differ significantly according to multiple comparison by Duncan’s test.

For all variables examined in vitro, except for average root length, Kondor microplants lagged behind in growth, as compared with Nevsky microplants (Fig. 1). The shoot length of Nevsky microplants was promoted by all inoculation treatments (see Fig. 1, a). The microplants inoculated with A. baldaniorum Sp245 alone were 18.9 % taller than the control – the maximum height among the treatments examined. In Kondor, all inoculation treatments suppressed shoot length, except for the treatment with A. baldaniorum Sp245, in which the plants did not differ from the control

In Nevsky, all inoculation treatments increased the number of nodes per shoot (see Fig. 1, b), except for inoculation with O. cytisi IPA7.2, when plant length did not differ from that in the control. The Nevsky microplants inoculated with A. baldaniorum Sp245 had 11.6 % more nodes than did the controls. The microplants inoculated sequentially with A. baldaniorum Sp245 (day 0) and O. cytisi IPA7.2 (day 15) had 5 % more nodes on their shoots than did the controls. The microplants inoculated simultaneously with A. baldaniorum Sp245 (day 15) and O. cytisi IPA7.2 (day 15) had 10.5 % more nodes than did the controls. The values for the Kondor microplants inoculated with A. baldaniorum Sp245 alone and sequentially with A. baldaniorum Sp245 (day 0) and O. cytisi IPA7.2 (day 15) were at the control level. The other inoculation treatments decreased the number of nodules on Kondor microplants.

In Nevsky, the average root length (see Fig. 1, c) increased by 4 % with A. baldaniorum Sp245 and by 3.7 % with O. cytisi IPA7.2, as compared with the control. However, sequential inoculation with A. baldaniorum Sp245 (day 0) and O. cytisi IPA7.2 (day 15) suppressed root length – it was 4.3 % lower than the control value. In Kondor, all inoculation treatments inhibited root length

In both cultivars, the number of roots (see Fig. 1, d) increased after both inoculation with A. baldaniorum Sp245 alone and sequential inoculation with A. baldaniorum Sp245 (day 0) and O. cytisi IPA7.2 (day 15). With A. baldaniorum Sp245, both Nevsky and Kondor had 12.5 % more roots than did the control. The Nevsky microplants inoculated sequentially with A. baldaniorum Sp245 (day 0) and O. cytisi IPA7.2 (day 15) had 6.3 % more roots than did the control. The Kondor microplants inoculated sequentially with A. baldaniorum Sp245 (day 0) and O. cytisi IPA7.2 (day 15) had 26.7 % more roots than did the control.

Thus, inoculation with A. baldaniorum Sp245 alone and sequential inoculation with A. baldaniorum Sp245 (day 0) and O. cytisi IPA7.2 (day 15) had positive effects on the Nevsky microplants. The shoot length, the number of nodes per shoot, and the number of roots increased, whereas the average root length decreased.

Identification of bacteria on roots of potato microplants in vitro

**Fig. 2.**
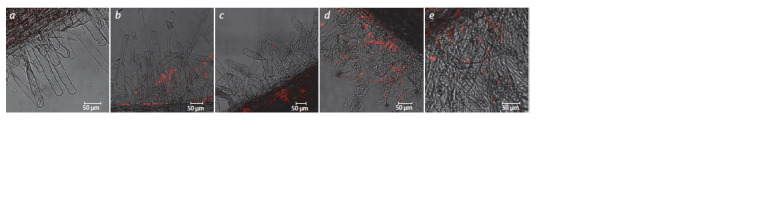
Identification of bacteria on roots of potato microplants by immunofluorescence confocal microscopy: a, control (no inoculation), antibodies to A. baldaniorum Sp245 and antibodies to O. cytisi IPA7.2; b, inoculation with A. baldaniorum Sp245, antibodies to A. baldaniorum Sp245; c, coinoculation with A. baldaniorum Sp245 and O. cytisi IPA7.2, antibodies to A. baldaniorum Sp245; d, coinoculation with A. baldaniorum Sp245 and O. cytisi IPA7.2, antibodies to O. cytisi IPA7.2; e, inoculation with O. cytisi IPA7.2, antibodies to O. cytisi IPA7.2.

Immunofluorescence analysis of the Nevsky roots by using confocal microscopy showed that both strains interacted successfully with plant cells (Fig. 2).

Both strains were detected on roots after both inoculation with pure cultures and coinoculation. Both strains were present in coinoculation treatments, which indicates that there was no antagonism between them and that neither strain had any advantage over the other in interacting with root cells.

Effect of bacteria on adaptation of potato microclones ex vitro

**Fig. 3.**
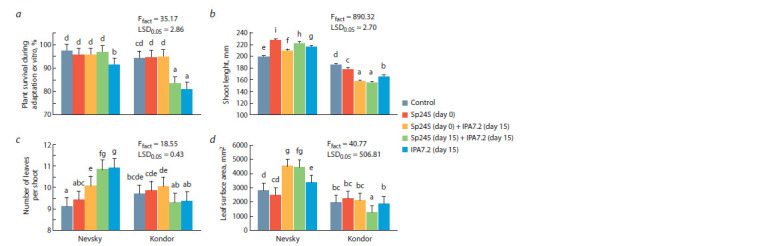
Effect of inoculation with A. baldaniorum Sp245 and O. cytisi IPA7.2 on morphological variables of potato microplants during plant adaptation ex vitro: a, plant survival during adaptation ex vitro; b, shoot length; c, number of leaves per shoot; d, leaf surface area.

The survival ability of the microplants formed in vitro in soil-filled vessels under phytochamber conditions (ex vitro stage) was high (more than 80 %) (Fig. 3, a). In Nevsky, the survival ability decreased by 6 %, as compared with the control, only after inoculation with O. cytisi IPA7.2 alone. In Kondor, the survival ability decreased by 11 % after coinoculation with A. baldaniorum Sp245 (day 15) and O. cytisi IPA7.2 (day 15) and by 14 % after inoculation with O. cytisi IPA7.2 alone.

Under ex vitro conditions, we found significant genotype effects on all variables examined. The Nevsky cultivar formed larger shoots with more large leaves than the Kondor cultivar (see Fig. 3).

In Nevsky, all inoculation treatments promoted shoot length (see Fig. 3, b). With A. baldaniorum Sp245 alone, shoot height increased by 14 %, as compared with the control; with A. baldaniorum Sр245 (day 0) and O. cytisi IPA7.2 (day15) inoculated sequentially, by 5 %; with A. baldaniorum Sp245 (day 15) and O. cytisi IPA7.2 (day 15) inoculated simultaneously, by 11.5 %; with O. cytisi IPA7.2 alone, by 8 %. In Kondor, all inoculation treatments suppressed shoot length (by 4 to 16 %).

The number of leaves per shoot (see Fig. 3, c) did not differ from the control value in any of the experimental treatments in Kondor. In Nevsky, on the contrary, all inoculation treatments promoted this variable, except for the use of A. baldaniorum Sp245 alone, in which no significant differences from the control were found. The Nevsky plants inoculated sequentially with A. baldaniorum Sр245 (day 0) and O. cytisi IPA7.2 (day 15) formed 10.5 % more leaves than did the control plants. The Nevsky plants inoculated simultaneously with A. baldaniorum Sp245 (day 15) and O. cytisi IPA7.2 (day 15) formed 18.7 % more leaves than did the control plants. The Nevsky plants inoculated with O. cytisi IPA7.2 alone had 19.4 % more leaves than did the control plants.

In Kondor, the leaf surface area (see Fig. 3, d) did not differ from the control value in any treatment except the simultaneous inoculation with A. baldaniorum Sp245 (day 15) and O. cytisi IPA7.2 (day 15), in which a 36.6 % negative effect was recorded. In Nevsky, the leaf surface area was promoted with O. cytisi IPA7.2 alone and with A. baldaniorum Sp245 coinoculated with O. cytisi IPA7.2 (in both coinoculation treatments, the leaf surface area was 60 % larger than that in the control). The Nevsky plants inoculated with O. cytisi IPA7.2 alone had larger leaves (by 19 %) than did the control plants.

Thus, the effects of microplant inoculation under in vitro conditions and during ex vitro adaptation depen-ded significantly on the plant genotype. In Nevsky, all variables were promoted with O. cytisi IPA7.2 alone and in combination with A. baldaniorum Sp245. In Kondor, the effect was negative, or the plants did not differ from the control ones.

Effect of bacteria on microplant growth in the soil-based greenhouse and on minituber yield

**Fig. 4.**
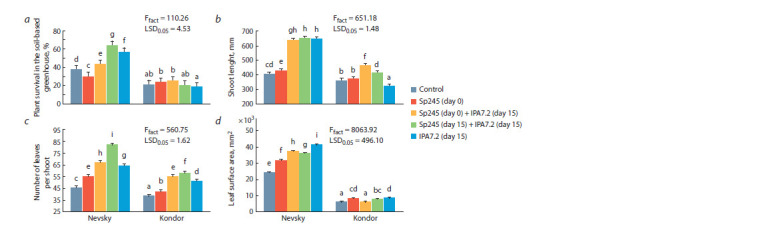
Effect of inoculation with A. baldaniorum Sp245 and O. cytisi IPA7.2 on variables of potato microplants grown in the soil-based greenhouse: a, plant survival in the soil-based greenhouse; b, shoot length; c, number of leaves per shoot; d, leaf surface area.

The survival ability of plants in the soil-based greenhouse was significantly lower than that in the vessels under controlled conditions (Fig. 4, a), because environmental factors were uncontrolled and depended on the surrounding milieu. In Nevsky, survival ranged from 30 to 64 %; in Kondor, it was even lower – 18.33 to 25 %. In Nevsky, plant survival in the soil-based greenhouse was promoted by inoculation with O. cytisi IPA7.2 alone (by 1.5 times) and with O. cytisi IPA7.2 combined with A. baldaniorum Sp245 (by 1.2 and 1.7 times). In Kondor, the inoculation results did not differ from the control values.

As in the previous stages, the Kondor cultivar had significantly less green matter than did the Nevsky cultivar. Under greenhouse conditions, the positive effect of inoculation was stronger than in the previous growing stages (see Fig. 4). In Kondor, shoot length was suppressed by 11 % only after inoculation with O. cytisi IPA7.2 alone. In Kondor, no significant effects were observed in two experimental treatments: A. baldaniorum Sp245 alone (shoot length) and A. baldaniorum Sp245 (day 0) combined with O. cytisi IPA7.2 (day 15) (leaf area). In the other treatments, inoculation led to positive effects.

In both cultivars, shoot length (see Fig. 4, b) was promoted by sequential inoculation with A. baldaniorum Sp245 (day 0) and O. cytisi IPA7.2 (day 15) (by 57.1 and 27.5 %, respectively) and by simultaneous inoculation (day 15) (by 60.6 and 13.8 %, respectively).

In both cultivars, the leaf number (see Fig. 4, c) was promoted the most after simultaneous inoculation with A. baldaniorum Sp245 (day 15) and O. cytisi IPA7.2 (day 15) (by 80.5 and 51.1 %, respectively).

In both cultivars, the leaf surface area (see Fig. 4, d) increased in most inoculation treatments, but the increase was greatest with O. cytisi IPA7.2 alone (by 71.0 % in Nevsky and by 41.0 % in Kondor).

**Fig. 5.**
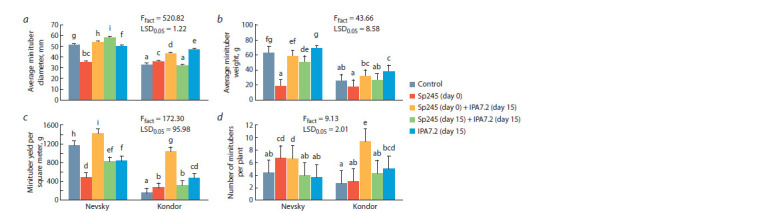
Effect of inoculation with A. baldaniorum Sp245 and O. cytisi IPA7.2 on minituber yield in potato plants grown in the soil-based greenhouse: a, average minituber diameter; b, average minituber weight; c, minituber yield per square meter; d, number of minitubers per plant.

Tuber size was promoted the most with O. cytisi IPA7.2 alone (in Kondor) and with A. baldaniorum Sр245 (day 0) and O. cytisi IPA7.2 (day 15) inoculated sequentially (in both cultivars) (Fig. 5a, b). In Kondor, the larger minituber diameter was increased the most with O. cytisi IPA7.2 alone (by 41.9 %), and in Nevsky, it was increased the most with A. baldaniorum Sp245 (day 15) and O. cytisi IPA7.2 (day 15) inoculated simultaneously (by 12.5 %).

In most experimental treatments, the weight of minitubers (see Fig. 5, b) did not differ from that in the control. In Nevsky, tuber weight was suppressed after inoculation with A. baldaniorum Sp245 alone (by 70.5 %) and after simultaneous inoculation with A. baldaniorum Sp245 (day 15) and O. cytisi IPA7.2 (day 15) (by 20.5 %). In Kondor, tuber weight increased by 48.7 % after inoculation with O. cytisi IPA7.2 alone (day 15). Thus, in Kondor, both size and weight of minitubers were promoted by O. cytisi IPA7.2.

In Kondor, the minituber yield was lower than that in Nevsky, in agreement with the morphometric variables in all previous stages (see Fig. 5, c). Yet, in the higher-yielding Nevsky cultivar inoculation increased the minituber yield to a lesser extent than in the lower-yielding Kondor cultivar. The positive effect of microplant inoculation in vitro was maximal after sequential inoculation with A. baldaniorum Sp245 (day 0) and O. cytisi IPA7.2 (day 15). In this inoculation treatment, the yield of minitubers per square meter increased by 11.1 % in Nevsky and 6.8-fold in Kondor.

In these experiments, between 2.67 and 9.33 tubers were obtained per plant (see Fig. 5, d). The number of tubers per plant did not differ significantly between cultivars. The sequential inoculation with A. baldaniorum Sp245 (day 0) and O. cytisi IPA7.2 (day 15) affected Nevsky and Kondor 3.5- and 1.5-fold, respectively. In Nevsky, the number of minitubers per plant was also increased with A. baldaniorum Sp245 alone. In Kondor, the number of minitubers per plant was also increased with O. cytisi IPA7.2 alone (1.9-fold).

Thus, sequential inoculation in vitro of microplants with A. baldaniorum Sp245 (day 0) and O. cytisi IPA7.2 (day 15) significantly increased the weight and number of minitubers, which constitute healthy and unconventional planting material

## Discussion

Generation of healthy planting material is important in potato production technology. The clonal micropropagation of pathogen-free plants by growing apical meristems in vitro is obligatory in potato seed production. The effectiveness of this method can be increased by using rhizosphere bacteria. The data in the literature indicate that bacteria promote plant growth at all stages, including growth in vitro and in vivo, and they also promote the adaptive ability of microplants planted in nonsterile settings ex vitro (Oswald et al., 2010; Belimov et al., 2015; Santiago et al., 2017; Soumare et al., 2021).

Our previous studies have shown that the associative rhizospheric bacteria A. baldaniorum Sp245 (Tkachenko et al., 2015) and O. cytisi IPA7.2 (Burygin et al., 2019) can be used to promote the growth of potato microplants in vitro and ex vitro. The ability of Azospirillum bacteria to promote potato growth and productivity, including in the seed production system, is well known (Naqqash et al., 2016; Kargapolova et al., 2020; Tkachenko et al., 2021). The efficacy of use of these bacteria is higher in vitro (optimal conditions) but is lower when plants are grown in the field (Bacilio et al., 2017).

Our results also show that as compared to the other treatments, inoculation with A. baldaniorum Sp245 alone better stimulated the growth of Nevsky microplants in vitro (optimal conditions) than it did ex vitro or in soil under greenhouse conditions. A. baldaniorum Sp245 was isolated from wheat roots (Baldani et al., 1983; Ferreira et al., 2020) and is a model for many studies. Our data show that this strain has a high ability to produce the plant hormone indole-3-acetic acid, which explains its promotion of the growth of microplant roots (Kargapolova et al., 2020).

O. cytisi IPA7.2, which we isolated directly from potato roots and which is native to the soils of Saratov Region, is more resistant to stress than Azospirillum (Burygin et al., 2017, 2019). This strain withstands large fluctuations in temperature and high salt and herbicide, which explains its ability to protect plants from stress, including osmotic stress (Evseeva et al., 2019).

The effect of bacterial inoculation on the formation and linear growth of plant organs (shoots and roots) depends on the hormonal balance existing in the plant at the moment. This balance is determined by genetic features, environmental factors, and the changes that specific strains cause in it (Arkhipova et al., 2020). Therefore, the promotion of shoot growth does not always coincide with that of root growth, and the effect of different strains may differ for different plant genotypes.

Combining different strains (e. g., azospirilla with other microsymbionts) for plant inoculation is considered promising owing to the possible synergistic effect and to the greater stability of the multicomponent system (Panahyan-e-Kivi et al., 2016; Trdan et al., 2019; Gavilanes et al., 2020). But in coinoculation, the compatibility of different strains and their ability to coinhabit plants without causing antagonism is important (O’Brien, Harrison, 2021). The efficacy of inoculation depends on plant genotype, development stage, and external and internal conditions (Andreote et al., 2010).

Previous work by us has found that the inoculation stage depends on the characteristics of the strain used (Burygin et al., 2018). A. baldaniorum Sp245 cannot grow independently on a nutrient growth medium for microplants and therefore can be used for inoculation at any stage of microplant growth in vitro. O. cytisi IPA7.2 can grow intensely on a nutrient growth medium for microplants and therefore can be used for inoculation only in the second half of the culturing period. Therefore, we examined two options for coinoculating microplants with A. baldaniorum Sp245 and O. cytisi IPA7.2: simultaneous inoculation (day 15 of growth) and sequential inoculation (A. baldaniorum Sp245 on day 0 and O. cytisi IPA7.2 on day 15 of growth). Our results show that A. baldaniorum Sp245 and O. cytisi IPA7.2 can be simultaneously present on potato roots without being antagonistic to each other (see Fig. 2). Synergistic effect, however, was not observed in all treatments, and it depended on the growth stage and the potato cultivar. Under in vitro conditions (see Fig. 1), the effects of the coinoculation treatments on most variables were not greater than the effect of each strain used separately, including A. baldaniorum Sp245, which was a good promoter of the growth of the Nevsky microplants.

In coinoculation, the adaptation ability of the microplants under favorable laboratory conditions at the stage of planting ex vitro (see Fig. 3) remained at the control level or at the level of the effects produced by the strains separately. But under the stressful conditions of the soil-based greenhouse, including poorly controlled environmental factors, the protective effect of inoculation was more pronounced (see Fig. 4), at least in Nevsky. In particular, after simultaneous inoculation, the survival ability of the Nevsky plants increased the most (by 71 %) – an effect greater than the positive effect of inoculation with O. cytisi IPA7.2 alone by almost 20 %.

Positive effect of coinoculation on the number and area of leaves was observed for the Nevsky cultivar during adaptation ex vitro (see Fig. 3), and the effect on the leaf area was synergistic. The promoting effect of inoculation was maximal under unfavorable greenhouse conditions (see Fig. 4), in agreement with the data of Cesari et al. (2019), who reported increased plant tolerance to stress under the influence of inoculation, including coinoculation with a bacterial consortium containing azospirilla. Coinoculation promoted the growth variables of both cultivars at the same level or even greater than did inoculation with O. cytisi IPA7.2 alone.

The efficacy of the whole technology of production of healthy potato planting material ultimately depends on the yield of minitubers. In seed breeding, it is not so much the weight of minitubers that matters as it is their number on plants, because the number of minitubers per plant determines the coefficient and rate of seed multiplication. The effect of inoculation on minituber production was particularly strong and was evident in both cultivars (see Fig. 5). The average minituber size changed nonsignificantly, but the number of minitubers on the plants increased significantly after sequential inoculation with A. baldaniorum Sp245 (day 0) and O. cytisi IPA7.2 (day 15). In Nevsky, the yield of minitubers was increased 1.5-fold; in Kondor, 3.5-fold. In Kondor, the sequential inoculation had a synergistic effect, as compared with the effects of the strains used separately. Similar synergistic effect on the yield of minitubers per square meter was noted in both cultivars in the same inoculation treatment.

The effects of sequential inoculation with A. baldaniorum Sp245 (microcuttings, day 0) and O. cytisi IPA7.2 (day 15) differed from those of simultaneous inoculation (day 15) at different stages of microplant growth. However, considering the promoting effect of A. baldaniorum Sp245 in vitro and the final yield of minitubers, sequential inoculation can be regarded as preferable.

## Conclusion

Analysis of the experimental data shows that the bacterial strains A. baldaniorum Sp245 and O. cytisi IPA7.2, both individually and in combination, had a positive effect on potato microplants. This effect manifested itself differently at different stages of plant growth. The maximal positive effect of inoculation in vitro was that on the number of adventitious roots; the number and area of leaves (during plant adaptation ex vitro); and the weight of minitubers and all variables for the vegetative portion of shoots (during plant growth in the soil-based greenhouse). The two strains were not antagonistic to each other. The growth-promoting effect of the bacteria depended significantly on the potato genotype. The positive effect of the interstrain interaction was maximal when plants were grown in the open ground. The strains A. baldaniorum Sp245 and O. cytisi IPA7.2, separately and in combination, can be recommended as inoculants for in vitro-grown potato microplants in potato clonal micropropagation to produce healthy planting material

## Conflict of interest

The authors declare no conflict of interest.
